# AN MHEALTH BEHAVIORAL INTERVENTION FOR PROMOTING PHYSICAL ACTIVITY AND SLEEP IN COMMUNITY-DWELLING OLDER ADULTS

**DOI:** 10.1093/geroni/igz038.603

**Published:** 2019-11-08

**Authors:** Junxin Li, Sarah Szanton, Minhui Liu, Nada Lukkahatai, Junxin Li, Nalaka Gooneratne

**Affiliations:** 1 Johns Hopkins School of Nursing, Baltimore, Maryland, United States; 2 Johns Hopkins University School of Nursing, Baltimore, Maryland, United States; 3 John Hopkins University, Baltimore, Maryland, United States; 4 University of Pennsylvania, Philadelphia, Pennsylvania, United States

## Abstract

Evidence suggests physical activity (PA) improves sleep in older adults. This study examined the preliminary effect of a personalized mHealth behavioral intervention on PA and sleep in older adults. We conducted a randomized controlled pilot trial in 21 community-dwelling older adults with sleep complaints. The 24-week mHealth behavioral intervention included a 2-hour in person training session, personalized exercise prescription, real time PA self-monitoring, interactive prompts, phone consultation, and weekly financial incentives. PA and sleep were measured objectively using Actiwatch 2.0 and subjectively using questionnaires. Peripheral blood was drawn for measuring Plasma inflammatory biomarkers [interleukin 1β, 6, 8, Tumor Necrosis Factor- alpha (TNF-α), and c-reactive protein (CRP)]. Data were collected at baseline, 8-week, 16-week, and post intervention. Repeated measures ANOVA (time*group) was used to examine differences of PA and sleep across times between the two groups. Majority of participants are women (71.4%) with mean age of 73.7 (SD = 6.9). Repeated measure ANOVA showed significant (p <0.05) improvement of objective and subjective PA, objective nocturnal sleep duration, self-report sleep quality (measured by Pittsburg Sleep Quality Index and Insomnia Severity Index) and decreasing of sedentary time over times in the intervention group (n=11), compared to the control group. The intervention group showed significant reduction of plasma TNF-α and CRP levels at 16-week and post intervention. Interventions combining personalized PA and mHealth strategies may positively affect physical activity and sleep in older adults. A larger study is needed to test the efficacy of this intervention and the mechanisms associated with it.

